# Equivalence of Anteroposterior and Frog-Leg Lateral Radiographs in Measuring Acetabular Index in Children

**DOI:** 10.21203/rs.3.rs-9861932/v1

**Published:** 2026-06-12

**Authors:** Atsuhiko Handa, Pedro H. Faria, Carter R. Petty, Andy Tsai, Michael B. Millis, Sarah D. Bixby

**Affiliations:** Boston Children’s Hospital; Boston Children’s Hospital; Boston Children’s Hospital; Boston Children’s Hospital; Boston Children’s Hospital; Boston Children’s Hospital

**Keywords:** developmental dysplasia of the hip, acetabular index, frog-leg lateral radiograph, pelvic positioning, radiation reduction

## Abstract

**Background:**

Developmental dysplasia of the hip (DDH) is one of the most common pediatric musculoskeletal conditions, with an incidence of 1 in 1,000 live births. The acetabular index (AI), measured on anteroposterior (AP) radiographs, is the primary radiographic tool for DDH diagnosis and monitoring. However, its accuracy is critically dependent on pelvic positioning; suboptimal studies frequently require repeat imaging, resulting in additional radiation exposure for the child.

**Objective:**

To determine whether frog-leg lateral (FL) radiographs are interchangeable with AP radiographs for AI measurement in children being evaluated for suspected DDH.

**Methods:**

In this retrospective, IRB-approved study, bilateral AP and FL hip radiographs from 100 pediatric patients (aged 6–24 months) were evaluated at a single tertiary pediatric center. Only AP studies meeting strict pelvic positioning criteria (rotation index 0.5–2.0; tilt index 0.9–1.4) were included. Four readers, including three pediatric radiologists and a pediatric orthopedic surgeon, independently measured bilateral AI on each view on two separate sessions, with a 4-week washout between sessions. Interchangeability was assessed using the Individual Equivalence Index (IEI) with a 3° margin. Reliability was assessed with intraclass correlation coefficients (ICC).

**Results:**

The √IEI was 2.07°, with a one-sided 95% upper confidence bound of 2.34°, both below the 3° interchangeability margin. Intra-reader ICCs ranged from 0.845 to 0.898 across both views. Three of four readers showed slightly higher repeatability on the FL view. Inter-reader ICC was 0.783 (95% CI: 0.720–0.833) for AP and 0.807 (95% CI: 0.744–0.854) for FL.

**Conclusions:**

AI measurements from FL radiographs are interchangeable with those from AP radiographs. When the AP view is suboptimally positioned, the FL view may be used for AI measurement, potentially eliminating repeat imaging and reducing radiation exposure in this pediatric population.

## Introduction

Developmental dysplasia of the hip (DDH) encompasses a spectrum of disorders ranging from mild acetabular dysplasia, with or without instability, to frank dislocation of the hip joint (1–3). The etiology is multifactorial, with risk factors including breech positioning, female sex, being firstborn, and positive family history. While mild abnormalities in early infancy may resolve spontaneously, persisting hip deformity with instability can progress to early-onset osteoarthritis and functional disability. With an incidence of approximately 1 in 1,000 live births, the DDH spectrum is one of the most common pediatric musculoskeletal conditions. Early diagnosis and treatment are essential for restoring normal hip anatomy and preventing long-term sequelae. The goal of treatment is to maintain concentric positioning of the femoral head within the acetabulum to promote normal joint development.

Ultrasound is the gold standard imaging tool for screening and diagnosis of DDH in infants under 4–6 months of age, as it allows direct visualization of the joint structures without ionizing radiation. However, as the acetabular roof ossifies after 4–6 months of age, plain radiography plays a central role in the diagnosis and follow up of DDH. The acetabular index (AI), measured on anteroposterior (AP) pelvic radiographs, is the most widely validated radiographic measure of acetabular morphology (4). It is defined as the angle between Hilgenreiner line (connecting the inferior-most points of bilateral ilia at the triradiate cartilages, also called the Yamamuro (Y) line) and the slope of the acetabular roof. An AI value greater than 30° in neonates is considered indicative of DDH, with the normal acetabulum becoming progressively less shallow with age (4, 5). Identifying the exact slope of the acetabular roof remains a point of debate. The classic method of connecting the inferior-most point of the iliac bone and the lateral-most point of the ossified acetabulum (5) may underestimate AI. Kim et al. demonstrated that this lateral-most acetabular margin often represents the anterolateral portion of the acetabulum, whereas the lateral end of the sourcil indicates the lateral margin of the mid-superior portion of the acetabulum (6). Using the sourcil as the landmark can yield significantly higher AI values, underscoring the need for precise anatomic landmarks.

A critical challenge in AI measurement is its sensitivity to pelvic positioning during radiographic acquisition (4, 7), with suboptimally positioned radiographs commonly seen in clinical practice. Both pelvic rotation (around the craniocaudal axis) and pelvic tilt (around the left–right axis) alter measured AI values (8). For example, when the pelvis is rotated to the left, the AI on an AP radiograph increases on the right side and decreases on the left, with the change related to the degree of rotation. Pelvic tilt similarly affects AI, though its direction of effect has been reported variably in the literature (4, 7, 9). In real-world situations, a combination of rotation and tilt is common and can further compound these effects on measured AI values (8).

Tönnis established criteria for assessing pelvic position on AP radiographs, introducing the ratio of the bilateral obturator foramen horizontal widths as a gauge of pelvic rotation. Building on this foundation, Portinaro et al. (7) and van der Bom et al. (8) each recommended a rotation index of 0.5–2.0, corresponding approximately up to ± 5° of rotation. Criteria for assessing pelvic tilt in infants, however, were not well established until recently despite prior attempts (4, 7, 8). While several tilt quantification methods have been proposed and validated in adult populations (10–12), these cannot be reliably applied in children due to incomplete ossification of the relevant anatomic landmarks. Boniforti et al (13) and Yang et al. (9) addressed this gap by defining a tilt index applicable in infants. This index is defined as the ratio of the vertical distance between the upper border of the pubic symphysis and Hilgenreiner line to the vertical diameter of the obturator foramen. The acceptable range of this index recommended by Yang et al. is 0.9–1.4, corresponding approximately up to ± 6° of tilt (9). These metrics are unfortunately seldom used in clinical practice, and suboptimal AP pelvic radiographs remain a common clinical occurrence. Repeat imaging is frequently required, which exposes an already radiosensitive pediatric population to additional ionizing radiation and imposing further burden on families and healthcare systems (14).

The frog-leg lateral (FL) radiograph, obtained with the hips abducted and externally rotated, is often routinely acquired alongside the AP view. This radiographic view often demonstrates improved concentricity of the femoral head. While the AP view is the historical gold standard for AI measurement, the availability of the FL view makes it a potential alternative when AP positioning is poor. Hudak et al. suggested that FL radiographs are as reliable as AP radiographs for assessing acetabular morphology in the pediatric hip, albeit in a small cohort of patients with normal hips (15). If FL-based AI measurements are equivalent to AP-derived measurements in clinically obtained cases, including those with DDH, which tend to be slightly more challenging to measure, the FL view could serve as a surrogate, thereby eliminating the need for repeat imaging. The present study was therefore designed to formally test this hypothesis using a rigorous interchangeability framework in a setting that mimics clinical practice.

## Materials and Methods

### Study Design and Patient Population

**Figure 1:**
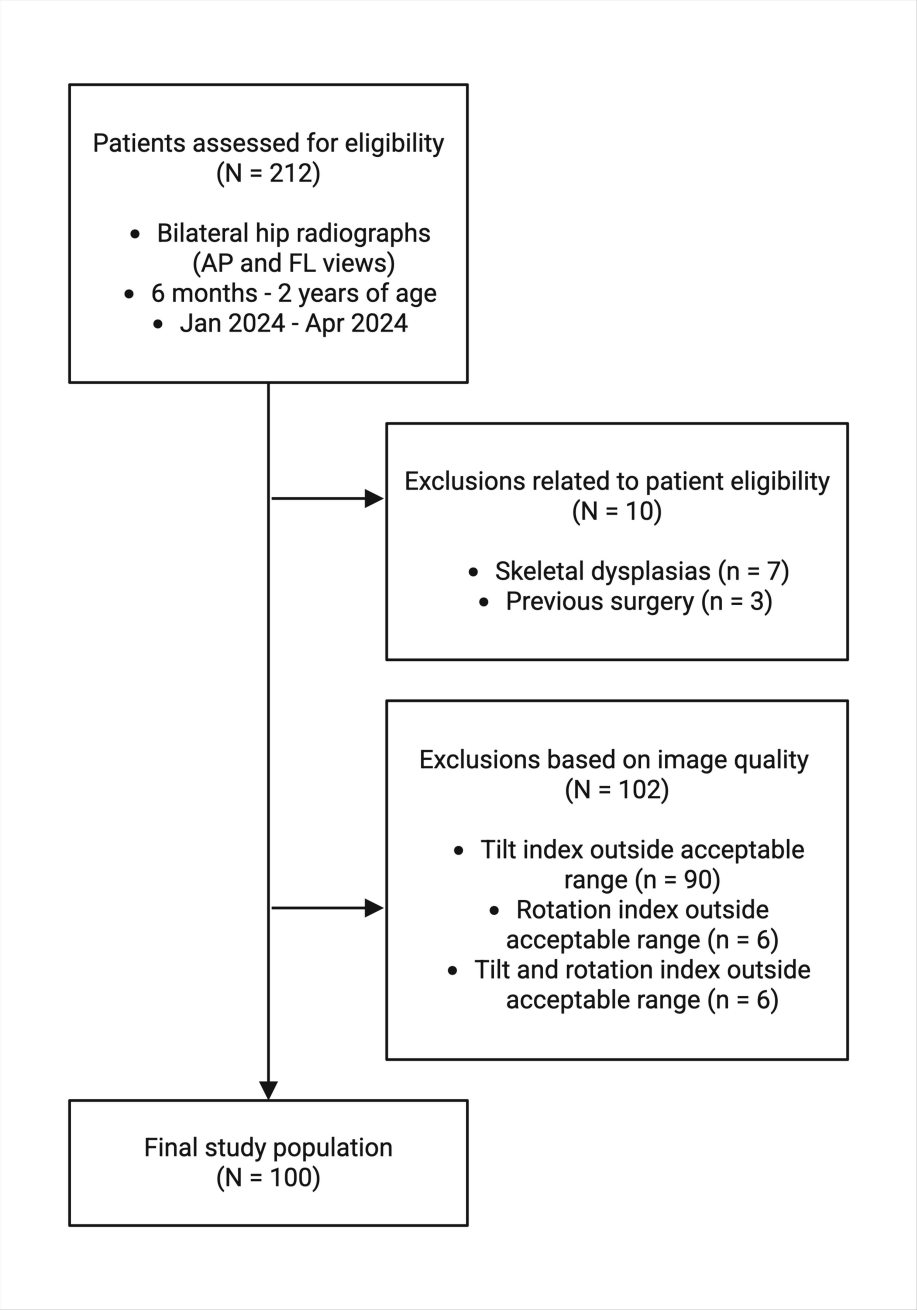
Flowchart illustrating patient selection.

This retrospective study was approved by the Institutional Review Board and was performed in accordance with the Health Insurance Portability and Accountability Act (HIPAA) at a single large tertiary pediatric center, using our institutional imaging archive. Inclusion criteria were patients aged 6–24 months with suspected DDH who had initial bilateral AP and FL studies obtained between January 2024 and April 2024. Exclusion criteria included a history of prior hip surgery or a diagnosis of skeletal dysplasia (Fig. 1). Follow-up radiographs performed within the study period were not included, so that patients were included only once in the study cohort.

**Figure 2:**
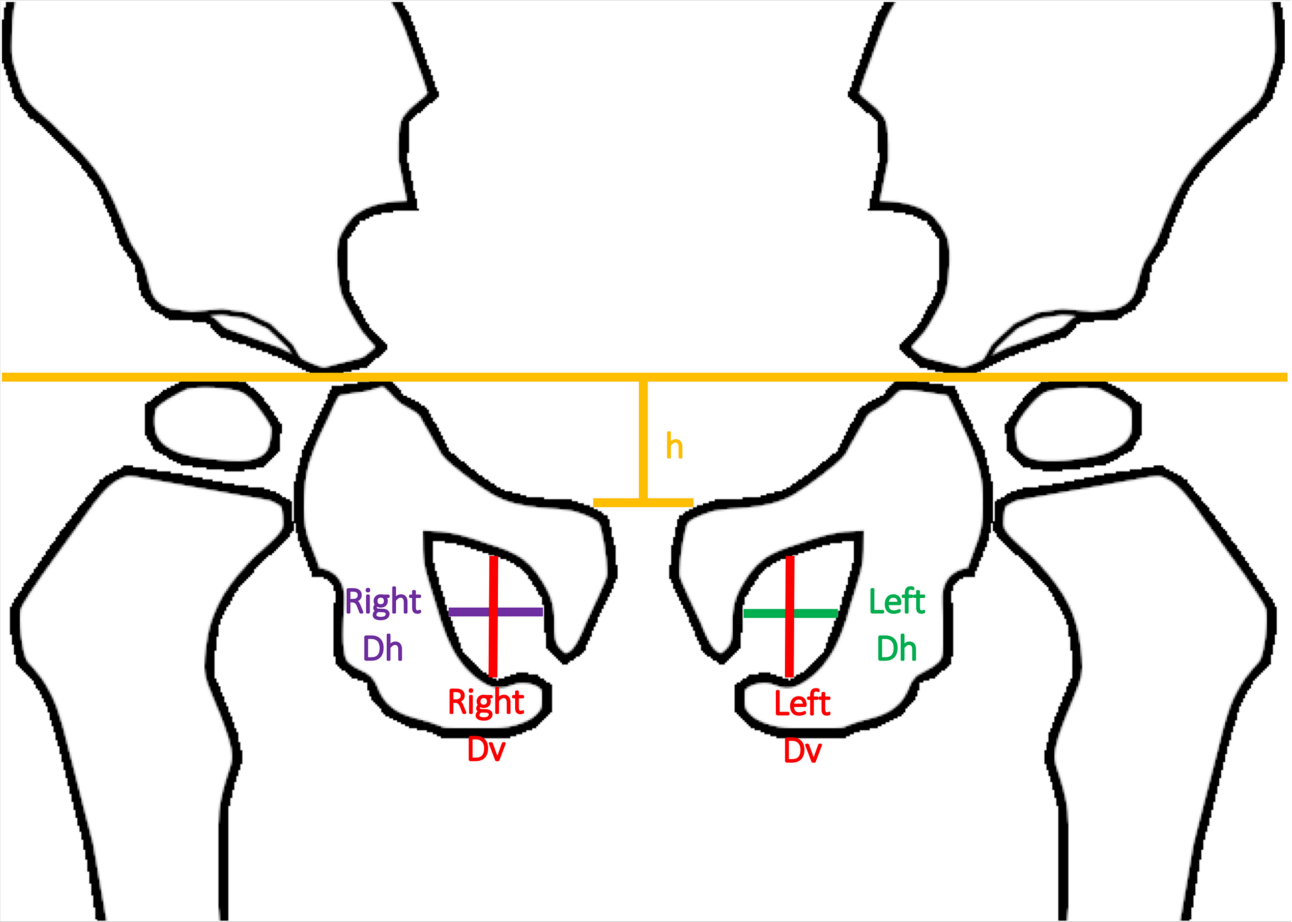
Schematic anteroposterior radiograph demonstrating key measurements used to assess pelvic rotation and tilt. The Hilgenreiner line connects the inferior margins of the bilateral triradiate cartilages. Pelvic rotation is quantified by dividing the horizontal diameter (Dh) of the right obturator foramen by that of the left obturator foramen (rotation index Rr = right Dh / left Dh). Pelvic tilt was assessed using the ratio of the vertical distance between the upper border of pubic symphysis and Hilgenreiner line (h) to the average vertical diameter of the obturator foramen (Dv = average of right Dv and left Dv) (tilt index Rt = h/Dv).

AP radiographic quality was assessed using the pelvic positioning criteria established by Portinaro et al (7), van der Bom et al (8) and Yang et al (9). Pelvic rotation was quantified by dividing the horizontal diameter (Dh) of the right obturator foramen by that of the left obturator foramen (rotation index Rr = right Dh / left Dh). Pelvic tilt was assessed using the ratio of the vertical distance between the upper border of pubic symphysis and Hilgenreiner line (h) to the average vertical diameter of the obturator foramen (Dv = average of right Dv and left Dv) (tilt index Rt = h/Dv) (Fig. 2). Only patients whose AP radiographs met predefined positioning criteria (rotation index 0.5–2.0; tilt index 0.9–1.4) were included. FL positioning variability was not controlled, reflecting the real-world clinical scenario in which the FL view is typically more readily obtained without motion. All curated studies were de-identified, assigned randomized identifiers, and stored in a secure research folder, with a corresponding worklist generated in the Picture Archiving and Communications System (PACS).

### Radiographic Measurement

AI was measured bilaterally on radiographic images using Hilgenreiner’s method. Measurements were performed using standard angle measurement tools in Synapse 7 PACS (Fujifilm Healthcare, Tokyo, Japan). All measurements were independently performed in a blinded fashion by four readers: three attending pediatric radiologists with 18 years, 16 years, and 4 years of post-fellowship experience, and one attending pediatric orthopedic surgeon specializing in hip disorders with 49 years of post-fellowship experience. Readers were blinded to each other’s measurements and to their own prior measurements. Each reader measured bilateral AI on both views in two separate sessions separated by a minimum four-week washout period to minimize recall bias. No pre-study session was conducted to reflect real-world measurement conditions.

### Statistical Analysis

The primary outcome was formal assessment of interchangeability between AP and FL AI measurements, using the Individual Equivalence Index (IEI) framework described by Obuchowski et al (16). This method evaluates whether the variability introduced by switching from one imaging modality to another exceeds that attributable to measurement variability within the same modality. Interchangeability was declared if the one-sided 95% upper confidence bound on the square root of the IEI (√IEI) fell below the pre-specified equivalence margin of 3°. This margin was selected by author consensus to correspond to 10% of the normal AI value at birth and to be stricter than the established thresholds for clinically acceptable AI measurement error reported by Portinaro et al. (± 5°) and van der Bom et al. (± 4.5°). The one-sided 95% upper confidence bound was estimated using a subject-level bootstrap procedure with 10,000 replications.

Secondary analyses of intra-reader repeatability and inter-reader agreement used two-way random-effects, absolute-agreement, single-measure intra-class correlation coefficients (ICC) with 95% confidence intervals. Intra-reader ICC was calculated separately for each reader and view by comparing Session 1 and Session 2 measurements across the same hips. For inter-reader ICC, Session 1 and Session 2 measurements were first averaged within each reader for each hip, yielding one reader-level measurement per hip. Inter-reader ICC was calculated separately for each view across all four readers. All statistical analyses were performed in R version 4.5.0 (R Foundation for Statistical Computing, Vienna, Austria) and Stata (StataCorp, College Station, TX, USA).

## Results

### Patient characteristics

The final study cohort consisted of 100 patients (200 radiographs), including 63 females and 37 males, with a median age at imaging of 1.0 years (interquartile range: 0.8–1.3 years). Of these, 37 patients carried a clinical diagnosis of DDH and underwent treatment. Table 1 provides additional patient characteristics.

### Equivalence of AP and FL Acetabular Index Measurements

The mean squared difference between AP view measurements made by random readers (within-modality variability) was 10.1°. The mean squared difference between AP and FL view measurements made by random readers (between-modality variability) was 14.4°. The estimated √IEI was 2.07°, indicating that switching from the AP view to the FL view produces AI measurement differences approximately 2.07° in excess of those expected from within-AP measurement variability alone. The one-sided 95% upper confidence bound, estimated by bootstrap procedure, was 2.34°. Both the point estimate and the upper confidence bound fell below the pre-specified 3° interchangeability margin (Table 2).

### Reader Agreement

Intra-reader ICC values for both views are summarized in Table 3. All four readers demonstrated high intra-reader repeatability on both views, with ICC values ranging from 0.845 to 0.898. Three of four readers showed slightly higher ICC values on the FL view compared to the AP view (Readers 2, 3, and 4; ΔICC: +0.025, + 0.042, and + 0.002, respectively). Reader 1 demonstrated identical repeatability across both views (ICC = 0.845 for both AP and FL).

Inter-reader ICC values across all four readers are presented in Table 4. The inter-reader ICC was 0.783 (95% CI: 0.720–0.833) for the AP view and 0.807 (95% CI: 0.744–0.854) for the FL view.

## Discussion

The principal finding of this study is that AI measurements obtained from FL radiographs are interchangeable with those from AP radiographs. To our knowledge, this represents the first formal interchangeability analysis comparing these two routinely obtained radiographic views for AI measurement in a pediatric population that includes patients with DDH, conditions that simulate real-world clinical practice.

These findings have immediate practical implications: a suboptimal AP view may not mandate repeat imaging when a contemporaneous FL view is available. The AP radiograph is the established gold standard for AI measurement; however, its quality is critically dependent on adequate pelvic positioning in young infants and toddlers—a population inherently prone to movement during radiographic acquisition. The practical challenge of obtaining acceptable AP radiographs in this age group is underscored by two observations: (1) the frequency with which repeat imaging is required in clinical practice due to suboptimal positioning, and (2) the high exclusion rate in our study. Specifically, 102 patients were excluded for failing to meet AP positioning criteria, approximately equal to the 100 who were included, suggesting that roughly one in two AP radiographs obtained in daily practice may be suboptimal for AI measurement by the quality metrics applied here. This high exclusion rate largely reflects the stringency of the tilt criteria. The tilt index of 0.9–1.4 recommended by Yang et al. resulted in exclusion of 90 patients on tilt alone. It is likely stricter than necessary compared to thresholds previously proposed in the literature, a question that warrants further investigation. While the AP view provides information not available on the FL view (such as assessment of femoral head concentricity in the neutral position), our results suggest that the routinely obtained FL view can serve as a direct substitute for AI measurement when the AP view is suboptimally positioned, potentially avoiding the additional radiation exposure and patient burden associated with repeat studies. The FL view may also be inherently less susceptible to positioning error, as achieving the abducted and externally rotated hip position likely restricts the degree of pelvic motion possible during image acquisition.

**Figure 3:**
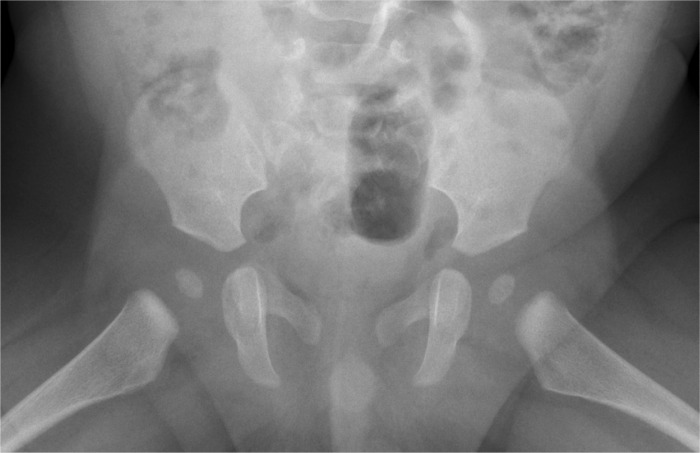
Representative FL radiograph demonstrating the acetabular sourcil as a single clear dense line, facilitating consistent landmark identification for AI measurement.

A secondary descriptive finding of interest is the slight trend toward higher reliability on the FL view. Three of four readers demonstrated higher intra-reader ICC on the FL view, and the inter-reader ICC was also higher for the FL view than the AP view (0.807 vs. 0.783). While these small differences should be interpreted cautiously, they invite hypotheses regarding the anatomic basis for more consistent landmark identification on the FL projection. The positioning of the hips in the FL position may project the acetabular sourcil in a plane that is more consistently identifiable. Indeed, there are many cases in which the sourcil appears as a single line on the FL view, potentially facilitating AI measurement (Fig. 3). Kim et al. demonstrated that sourcil configuration varies significantly with projection and patient age, and our findings suggest the FL projection may reduce some of this variability. This observation merits further investigation in a dedicated reproducibility study.

Several design elements strengthen the validity of this study. We applied strict, pre-specified AP quality criteria following the recommendations of Tönnis, Portinaro et al., van der Bom et al., and Yang et al., ensuring that AP reference measurements reflected acceptable pelvic alignment. The inclusion of four experienced readers across two clinical specialties enhances generalizability to the full care team that relies on AI measurements in daily practice. Blinded, repeated measurements with a minimum four-week washout interval minimized recall bias. The 3° equivalence margin was pre-specified rather than selected post-hoc and is stricter than thresholds previously reported in the literature. Finally, the IEI framework is specifically designed to address measurement interchangeability and provides appropriate analytic approach for the clinical question.

This study has several limitations. This was a single-center retrospective study at a tertiary pediatric referral center, and our findings may not be directly generalizable to community-based settings with different patient demographics, technologist training, or variable rates of FL radiograph acquisition. Because our cohort was restricted to patients aged 6–24 months, the findings cannot be extrapolated to neonates and children outside this age range, representing an opportunity for future study. The strict tilt criteria applied to AP view in this study might have excluded the range of clinically acceptable pelvic positioning encountered in daily practice, and future studies using broader or alternative quality thresholds may yield a more representative cohort. FL positioning variability was intentionally unmeasured, reflecting the clinical scenario of interest: using the FL view as acquired when the AP is suboptimal. Given this study design, we did not characterize the degree of FL positioning errors present in the sample. This study assessed measurement equivalence only and does not provide evidence that treatment decisions based on FL-derived AI values lead to equivalent clinical outcomes. Finally, while 100 patients with four blinded readers and two measurement sessions provide a robust dataset, a larger multicenter study would further confirm the generalizability of these findings.

## Conclusion

AI measurements obtained from FL radiographs are interchangeable with those from AP radiographs. Both views showed high and comparable intra- and inter-reader reliability, with the FL view demonstrating marginally superior performance across most readers. These findings support the use of the routinely obtained FL view as a direct substitute for AI measurement when the AP view is suboptimally positioned, with the potential to eliminate repeat radiographic studies and reduce radiation exposure in this pediatric population.

## Figures and Tables

**Table 1:** Patient characteristics

Characteristics	N = 100
Female sex	63 (63%)
Patient age (years) at the time of imaging	1.0 years [0.8–1.3] †
Diagnosis of DDH	37 (37%)

† Data is median and interquartile range

**Table 2:** Equivalence Analysis Results

Parameter	Value
Mean squared difference (AP vs. AP, same readers)	10.1°
Mean squared difference (AP vs. FL, same readers)	14.4°
√IEI – point estimate	2.07°
One-sided 95% upper confidence bound	2.34°
Pre-specified interchangeability margin	3.00°

IEI = Individual Equivalence Index; UCB = upper confidence bound; AP = anteroposterior; FL = frog-leg lateral. The pre-specified equivalence margin of 3° was established prior to analysis.

**Table 3:** Intra-Reader Repeatability: Intraclass Correlation Coefficients by Reader and Radiographic View

Reader	AP ICC (95% CI)	FL ICC (95% CI)
Reader 1	0.845 (0.799–0.882)	0.845 (0.800–0.880)
Reader 2	0.869 (0.830–0.900)	0.894 (0.852–0.923)
Reader 3	0.856 (0.808–0.892)	0.898 (0.867–0.922)
Reader 4	0.876 (0.827–0.910)	0.878 (0.835–0.909)

ICC = intraclass correlation coefficient; AP = anteroposterior; FL = frog-leg lateral. ICC calculated from Session 1 vs. Session 2 measurements within each reader.

**Table 4:** Inter-Reader Agreement: Intraclass Correlation Coefficients by Radiographic View

View	ICC (95% CI)
AP	0.783 (0.720–0.833)
FL	0.807 (0.744–0.854)

ICC = intraclass correlation coefficient; CI = confidence interval; AP = anteroposterior; FL = frog-leg lateral. ICC calculated across all four readers after averaging Session 1 and Session 2 measurements per reader.
